# Association Between Cardiovascular Disease and Complete Edentulism in U.S. Adults

**DOI:** 10.3390/jcm14176035

**Published:** 2025-08-26

**Authors:** Saud Alyahya, Basel Hamoud, Ali Alqattan, Masoud Almasoud, Yousef Almehjan, Rashed Alajmi, Hesham Alhazmi, Hend Alqaderi

**Affiliations:** 1 Kuwait Ministry of Health, Kuwait City 15462, Kuwait; dr.saudalyahya@gmail.com (S.A.); aalqattan95@gmail.com (A.A.); msalmasoud@outlook.co (M.A.); yousefalmehjan1@hotmail.com (Y.A.); rashedalhyyan@gmail.com (R.A.); 2 Department of Preventive Dentistry, Faculty of Dental Medicine, Umm Al-Qura University, Makkah 24382, Saudi Arabia; dr.h.a.alhazmi@gmail.com; 3 Department of Public Health, School of Dental Medicine, Tufts University, Boston, MA 02111, USA; hendalqaderi@gmail.com; 4 Dasman Diabetes Institute, Kuwait City 15462, Kuwait

**Keywords:** cardiovascular disease (CVD), edentulism, missing tooth, tooth loss, NHANES

## Abstract

(1) **Background:** Cardiovascular disease (CVD) and edentulism are major public health challenges with shared risk factors and overlapping inflammatory pathways. This study investigates the association between complete tooth loss and CVD. (2) **Methods:** Data were analyzed from the 2015–2018 National Health and Nutrition Examination Survey (NHANES) dataset using Poisson regression analysis to examine the relationship between CVD and complete edentulism, adjusting for age, sex, education, family income-to-poverty ratio, race/ethnicity, diabetes status, and BMI. Of the 11,287 participants, 1763 individuals (15.62%) were completely edentulous, and 9524 (84.38%) retained some or all of their dentition. (3) **Results:** Individuals with cardiovascular conditions, including myocardial infarction (PR = 1.55; 95% CI: 1.23–1.98), coronary heart disease (PR = 1.44; 95% CI: 1.13–1.85), congestive heart failure (PR = 1.58; 95% CI: 1.22–2.07), and stroke (PR = 1.46; 95% CI: 1.13–1.90), demonstrated a higher prevalence of complete edentulism compared to those without these conditions, after adjusting for key demographic and health-related confounders (*p* < 0.01). (4) **Conclusions:** These findings suggest a statistical association between CVD and complete edentulism in U.S. adults. However, due to the cross-sectional nature of this study, causal relationships cannot be inferred, and further longitudinal studies are needed to understand the bidirectional mechanisms between CVD and complete edentulism.

## 1. Introduction

Tooth loss, often resulting from periodontitis, is not only a reflection of poor oral hygiene or aging but also a potential predictor of cardiovascular morbidity and mortality [1,2,3]. According to the American College of Prosthodontics (ACP), an estimated 178 million Americans are missing at least one tooth, and approximately 40 million are completely edentulous [4]. Evidence shows that the loss of multiple teeth has been associated with various systemic health outcomes and chronic conditions [5,6]. These include hypertension [7], diabetes mellitus [8], peripheral arterial disease [9], cardiovascular diseases such as heart failure, stroke, and angina pectoris [10,11,12], overweight and obesity [13], chronic kidney disease [14], chronic obstructive pulmonary disease [15], dementia [16], depression [17], and cognitive decline [18].

Periodontitis and cardiovascular diseases (CVDs) frequently coexist and share common comorbidities, such as hypertension, diabetes, and dyslipidemia [19]. Recent evidence highlights that this association is increasingly recognized as a biologically plausible link, supported by findings of reciprocal inflammatory pathways, whereby both periodontal and cardiovascular diseases contribute to systemic immune activation [1,2,3,20]. Elevated levels of inflammatory mediators such as CRP, IL-6, and TNF-α have been implicated in both conditions, which may lead to endothelial dysfunction, vascular inflammation, and atherogenesis [1,2,3,20]. Moreover, periodontal pathogens like *Porphyromonas gingivalis* may invade vascular tissues and contribute to inflammation and atherogenesis [21]. Recent research also reveals that patients with CVDs and tooth loss reported greater pain, chewing difficulties, and psychosocial stress [22,23]. As a result, this may indirectly worsen cardiovascular outcomes by impairing diet, mental health, and medication adherence [22,23].

Beyond mechanistic pathways, a recent meta-analysis found that those with severe tooth loss (fewer than 10 teeth) had nearly double the risk of stroke, myocardial infarction, heart failure, and all-cause mortality [24]. Research has shown that the relationship between CVD and tooth loss appears to be bidirectional, emphasizing that CVD may worsen oral health, while poor oral health may contribute to CVD progression [25]. Individuals with CVD may experience diminished oral blood flow, altered salivary composition, and immune suppression due to both the disease itself and its pharmacological management [26,27,28].

Despite growing evidence of this link, many existing studies have relied on small or region-specific samples, limiting the generalizability of their findings to broader populations [22,29,30]. Moreover, there are few studies that control for socioeconomic and demographic variables while still demonstrating an independent association between CVD and complete edentulism [31,32,33]. In addition, while prior studies have proven a general association between CVDs and edentulism, limited evidence exists regarding how specific CVD subtypes such as heart failure, coronary heart disease, and stroke differ in their associations with complete tooth loss [29,34,35]. To address this gap, the present study aims to evaluate the association between CVD and complete edentulism using data from the 2015–2018 National Health and Nutrition Examination Survey (NHANES). We hypothesize that individuals with CVD are more likely to be completely edentulous, potentially due to shared inflammatory and vascular mechanisms.

## 2. Materials and Methods

### 2.1. Study Population 

This cross-sectional study utilized data from the 2015–2018 National Health and Nutrition Examination Survey (NHANES), a nationally representative program conducted by the Centers for Disease Control and Prevention (CDC) [36]. NHANES collects health, nutritional, and dental data through structured interviews, surveys, clinical examinations, and laboratory investigations, using a multistage, stratified, clustered sampling design to reflect the non-institutionalized U.S. population [36].

**Figure 1:**
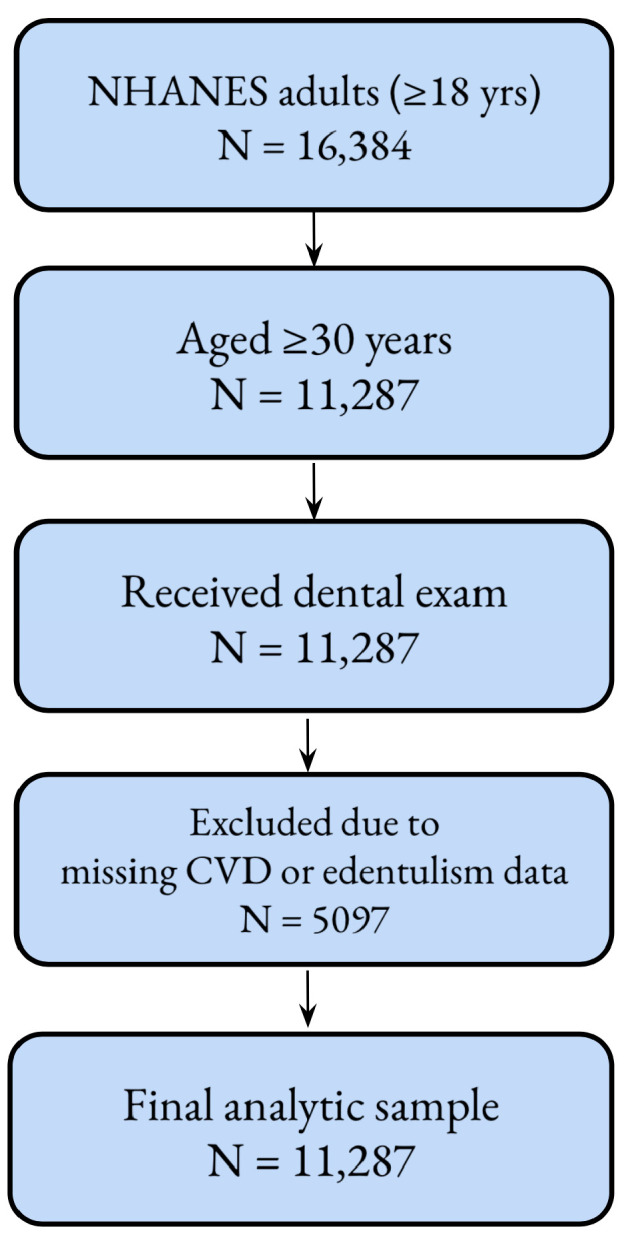
The flowchart of participant inclusion and exclusion criteria following STROBE guidelines.

A total of 16,384 adults (aged ≥18 years) participated in the 2015–2018 NHANES cycles. For this analysis, we restricted the sample to individuals aged ≥30 years who received a dental examination. Participants with missing data on complete edentulism, cardiovascular disease (CVD), or relevant covariates were excluded (*n* = 5097), resulting in a final analytic sample of 11,287 adults. A detailed flowchart describing the inclusion and exclusion process is presented in Figure 1.

All participants provided written informed consent. This study used publicly available, de-identified NHANES data and was therefore classified as secondary data analysis, exempt from Institutional Review Board (IRB) review. This study followed the STROBE (Strengthening the Reporting of Observational Studies in Epidemiology) guidelines. Nationally representative estimates were obtained by incorporating NHANES sample weights, strata, and primary sampling units (PSUs) in all analyses.

### 2.2. Definition of the Dependent Variable: Complete Tooth Loss

The primary outcome in this study was complete edentulism, defined as the absence of all natural permanent teeth, including third molars. This variable was coded as binary: “0” for individuals with any remaining dentition and “1” for those with complete tooth loss. Oral health assessments were conducted by licensed dentists at NHANES Mobile Examination Centers (MECs) using a standardized protocol. Examiners were trained and calibrated by the National Center for Health Statistics to ensure consistency. The presence or absence of teeth was determined through direct clinical examination rather than self-report. Details of the oral health protocol are available in the NHANES documentation [36].

According to the American Association of Oral and Maxillofacial Surgeons (AAOMS), edentulism refers to the loss of one or more functional teeth and is typically categorized as partial or complete, depending on the extent of tooth loss; common causes include dental caries, periodontal disease, and trauma [37]. For this analysis, only individuals with complete edentulism were classified as cases and compared to those with preserved dentition. This binary classification enabled the evaluation of the association between complete edentulism and CVD within a cross-sectional epidemiologic framework.

### 2.3. Description of Independent Variable: CVD

CVD conditions, including myocardial infarction, coronary heart disease, congestive heart failure, and stroke, were assessed through self-reported responses to standardized NHANES interview questions. Participants were asked whether a health professional had ever informed them of having any of these conditions. No clinical verification (e.g., medical records or diagnostic testing) was conducted as part of the NHANES protocol. These conditions were analyzed both individually and as a combined ‘heart disease’ category for broader analysis. CVD status was coded as a binary variable (Yes/No), with “Yes” indicating that participants reported at least one of the listed diagnoses and “No” indicating none. Although myocardial infarction (MI) is a clinical subtype of coronary heart disease (CHD), NHANES collects self-reported history of these conditions through separate questionnaire items. To preserve the original structure of the data and evaluate the specific associations with edentulism, we analyzed them as distinct variables.

### 2.4. Potential Confounding Variable 

Several demographic and health-related variables were included as potential confounders and adjusted for in the analysis. Age was categorized into three groups (19–44, 45–59, and ≥60 years) but was analyzed as a continuous variable. Sex was treated as a binary variable (male or female), and race/ethnicity was categorized as Non-Hispanic White, Non-Hispanic Black, Hispanic, Non-Hispanic Asian, and Other.

Education level was classified into three categories: less than high school, high school or GED, and more than high school education. Family income was expressed as a ratio to the federal poverty level (FPL) and categorized as <100% FPL, 100–199% FPL, 200–399% FPL, and ≥400% FPL.

Body mass index (BMI) was treated as a categorical ordinal variable and classified as underweight, normal weight, overweight, and obese. Diabetes status was determined based on the response to the question: “Have you ever been told by a doctor or health professional that you have diabetes or sugar diabetes?” Responses were grouped as “No” (no diabetes) and “Yes” (including borderline cases) to reflect the presence of diabetes or prediabetes.

This classification is consistent with prior studies using NHANES data [38,39] and was adopted to preserve model stability and account for the elevated health risk shared by individuals with diabetes and borderline diabetes.

### 2.5. Statistical Methods

Descriptive statistics were used to summarize the characteristics of the study population. Categorical variables were compared using chi-square tests, and continuous variables were compared using *t*-tests. To assess the association between complete edentulism and CVD, we used a weighted multivariate Poisson regression model with robust error variance, adjusting for age, sex, education level, race/ethnicity, BMI, family income-to-poverty ratio, and diabetes status. All analyses incorporated NHANES sampling weights, strata, and primary sampling units (PSUs) to account for the complex, multistage probability sampling design and to ensure nationally representative estimates. Prevalence ratios (PRs) and their corresponding 95% confidence intervals (CIs) were estimated. A *p* value of <0.05 was considered statistically significant. All analyses were performed using STATA 17.0 software (StataCorp LLC., College Station, TX, USA).

## 3. Results

Table 1 summarizes the weighted characteristics of the study population by complete edentulism status. Of the 11,287 participants, 1763 (15.62%) were completely edentulous. The proportion of complete tooth loss was higher among those with heart disease (1.76%) compared to those without (6.42%) (*p* < 0.0001). Participants with a history of myocardial infarction had a complete tooth loss rate of 0.78%, while the rate among those without was 7.42% (*p* < 0.0001). Those with coronary heart disease had a rate of 0.74% compared to 7.39% among those without (*p* < 0.0001). For individuals with congestive heart failure, the rate was 0.58%, while it was 7.61% among those without (*p* < 0.0001). Stroke was linked to an edentulism rate of 0.64%, compared to 7.61% in those without stroke (*p* < 0.0001). Gender distribution showed similar proportions: 6.65% in males and 6.78% in females (*p* = 0.53). Edentulism was highest among White participants (7.41%), followed by Black (6.70%), Hispanic (2.72%), Asian (2.10%), and Others (1.87%) (*p* = 0.00017). Age differences were observed, with higher rates among older age groups, ranging from 0.28% in participants aged 6–11 to 3.14% in those above 60 years (*p* < 0.0001). Participants with lower education levels (0–11 years) had an edentulism rate of 2.13%, compared to 1.35% among those with more than a high school education (*p* < 0.0001). Edentulism was most frequent in the lowest income group (<100% FPL) at 30.42%, and it decreased with higher income levels (*p* < 0.0001). Among BMI categories, edentulism was observed at 2.52% among obese individuals and 1.97% among those with normal BMI (*p* < 0.0001). Diabetes status showed an edentulism rate of 1.24% among participants with diabetes and 10.99% among those without, with no notable difference across groups (*p* = 0.0976).

Table 2 displays the results from the weighted multiple Poisson regression model showing the association between myocardial infarction and complete edentulism in the United States, 2015–2018. Individuals with a history of myocardial infarction had a higher prevalence of complete edentulism (PR = 1.55; 95% CI: 1.225–1.980; *p* = 0.001). Compared to individuals aged 30–44 years, those aged 45–59 (PR = 2.10; 95% CI: 1.558–2.848; *p* < 0.001) and those above 60 years (PR = 3.70; 95% CI: 2.576–5.326; *p* < 0.001) had a greater prevalence of complete edentulism. Higher levels of education were inversely associated with edentulism, with high school/GED (PR = 0.70; 95% CI: 0.580–0.859; *p* = 0.001) and more than high school education (PR = 0.46; 95% CI: 0.380–0.556; *p* < 0.001) showing reduced prevalence. Household income above the poverty level was also associated with lower prevalence of edentulism, including those at 200–399% FPL (PR = 0.58; 95% CI: 0.427–0.809; *p* = 0.002) and 400% + FPL (PR = 0.42; 95% CI: 0.294–0.609; *p* < 0.001). Compared to Hispanic individuals, the prevalence of edentulism was higher among Non-Hispanic Whites (PR = 1.44; 95% CI: 1.045–2.009; *p* = 0.027), Non-Hispanic Blacks (PR = 1.50; 95% CI: 1.109–2.051; *p* = 0.010), Non-Hispanic Asians (PR = 1.77; 95% CI: 1.198–2.624; *p* = 0.006), and those identified as Other race/ethnicity (PR = 2.17; 95% CI: 1.448–3.264; *p* < 0.001). Sex, diabetes status, and BMI categories were not statistically associated with complete edentulism in this model.

Table 3 displays the results from the weighted multiple Poisson regression model showing the association between heart disease and complete edentulism in the United States, 2015–2018. Individuals with heart disease had a higher prevalence of complete edentulism (PR = 1.53; 95% CI: 1.280–1.842; *p* < 0.001). Compared to adults aged 30–44 years, those aged 45–59 (PR = 2.07; 95% CI: 1.537–2.796; *p* < 0.001) and above 60 (PR = 3.52; 95% CI: 2.458–5.052; *p* < 0.001) demonstrated a higher prevalence. Higher educational attainment was associated with lower prevalence, including those with a high school/GED (PR = 0.71; 95% CI: 0.582–0.866; *p* = 0.001) and more than high school education (PR = 0.46; 95% CI: 0.382–0.559; *p* < 0.001). Participants with income levels at 200–399% FPL (PR = 0.59; 95% CI: 0.431–0.813; *p* = 0.002) and 400% + FPL (PR = 0.43; 95% CI: 0.301–0.619; *p* < 0.001) had lower prevalence of edentulism compared to those below 100% FPL. Compared to Hispanic individuals, higher prevalence was observed among Non-Hispanic Whites (PR = 1.41; 95% CI: 1.016–1.962; *p* = 0.040), Non-Hispanic Blacks (PR = 1.46; 95% CI: 1.073–1.997; *p* = 0.018), Non-Hispanic Asians (PR = 1.76; 95% CI: 1.189–2.629; *p* = 0.006), and those categorized as Other race (PR = 2.14; 95% CI: 1.413–3.242; *p* = 0.001). Sex, diabetes status, and BMI categories were not associated with complete edentulism in this model.

Table 4 displays the results from the weighted multiple Poisson regression model showing the association between coronary heart disease and complete edentulism in the United States, 2015–2018. Individuals with coronary heart disease had a higher prevalence of complete edentulism (PR = 1.44; 95% CI: 1.132–1.853; *p* = 0.004). Compared to adults aged 30–44 years, those aged 45–59 (PR = 2.12; 95% CI: 1.572–2.872; *p* < 0.001) and those above 60 (PR = 3.73; 95% CI: 2.598–5.375; *p* < 0.001) had a higher prevalence. Higher education levels were inversely associated with edentulism, including those with high school/GED (PR = 0.71; 95% CI: 0.585–0.884; *p* = 0.003) and more than high school education (PR = 0.46; 95% CI: 0.378–0.567; *p* < 0.001). Lower prevalence was also observed among individuals at 200–399% FPL (PR = 0.58; 95% CI: 0.425–0.809; *p* = 0.002) and 400% + FPL (PR = 0.42; 95% CI: 0.294–0.605; *p* < 0.001). Compared to Hispanic individuals, Non-Hispanic Whites (PR = 1.43; 95% CI: 1.025–2.001; *p* = 0.036), Non-Hispanic Blacks (PR = 1.51; 95% CI: 1.110–2.066; *p* = 0.010), Non-Hispanic Asians (PR = 1.77; 95% CI: 1.199–2.618; *p* = 0.005), and individuals of Other race/ethnicity (PR = 2.22; 95% CI: 1.468–3.356; *p* < 0.001) had higher prevalence of edentulism. Sex, diabetes status, and BMI categories were not associated with complete edentulism in this model.

Table 5 displays the results from the weighted multiple Poisson regression model showing the association between congestive heart failure and complete edentulism in the United States, 2015–2018. Individuals with congestive heart failure had a higher prevalence of complete edentulism (PR = 1.58; 95% CI: 1.216–2.067; *p* = 0.001). Compared to those aged 30–44 years, higher prevalence was observed among participants aged 45–59 (PR = 2.12; 95% CI: 1.577–2.868; *p* < 0.001) and above 60 (PR = 3.78; 95% CI: 2.612–5.482; *p* < 0.001). Higher education was associated with lower prevalence, including high school/GED (PR = 0.70; 95% CI: 0.574–0.864; *p* = 0.001) and more than high school (PR = 0.45; 95% CI: 0.374–0.559; *p* < 0.001). Participants with income at 200–399% FPL (PR = 0.58; 95% CI: 0.425–0.809; *p* = 0.002) and 400% + FPL (PR = 0.42; 95% CI: 0.295–0.607; *p* < 0.001) had lower prevalence of edentulism compared to those below 100% FPL. Compared to Hispanic individuals, higher prevalence was observed among Non-Hispanic Whites (PR = 1.45; 95% CI: 1.041–2.019; *p* = 0.029), Non-Hispanic Blacks (PR = 1.48; 95% CI: 1.087–2.026; *p* = 0.015), Non-Hispanic Asians (PR = 1.75; 95% CI: 1.185–2.602; *p* = 0.007), and individuals of Other race (PR = 2.25; 95% CI: 1.494–3.407; *p* < 0.001). Sex, diabetes status, and BMI categories were not associated with complete edentulism in this model.

Table 6 displays the results from the weighted multiple Poisson regression model showing the association between stroke and complete edentulism in the United States, 2015–2018. Individuals with a history of stroke had a higher prevalence of complete edentulism (PR = 1.46; 95% CI: 1.133–1.898; *p* = 0.005). Compared to those aged 30–44 years, adults aged 45–59 (PR = 2.13; 95% CI: 1.583–2.872; *p* < 0.001) and above 60 (PR = 3.80; 95% CI: 2.636–5.486; *p* < 0.001) had greater prevalence. Higher education was associated with lower prevalence, including high school/GED (PR = 0.70; 95% CI: 0.574–0.864; *p* = 0.001) and more than high school education (PR = 0.46; 95% CI: 0.378–0.560; *p* < 0.001). Lower prevalence was also observed among those at 200–399% FPL (PR = 0.58; 95% CI: 0.426–0.811; *p* = 0.002) and 400% + FPL (PR = 0.42; 95% CI: 0.296–0.607; *p* < 0.001). Compared to Hispanic individuals, higher prevalence was found among Non-Hispanic Whites (PR = 1.45; 95% CI: 1.046–2.027; *p* = 0.027), Non-Hispanic Blacks (PR = 1.49; 95% CI: 1.095–2.047; *p* = 0.013), Non-Hispanic Asians (PR = 1.78; 95% CI: 1.204–2.646; *p* = 0.005), and individuals classified as Other race (PR = 2.24; 95% CI: 1.480–3.404; *p* < 0.001). Sex, diabetes status, and BMI categories were not associated with complete edentulism in this model.

The prevalence rates among different CVD outcomes are presented in Table 7.

## 4. Discussion

The present study examined the association between CVD and complete edentulism in a large, nationally representative sample of U.S. adults. Our analysis indicated a positive relationship between complete edentulism and all major CVD types, including heart attack, coronary heart disease, congestive heart failure, and stroke. Although congestive heart failure showed the highest prevalence ratio, the overlapping confidence intervals suggest that differences across CVD types may not be statistically distinguishable. Therefore, caution is warranted when interpreting variations in the strength of these associations. This study is among the few to explore complete edentulism in relation to multiple CVD subtypes within a U.S. population while accounting for a wide range of demographic and health-related factors.

Population-based research has consistently reported associations between CVD and tooth loss or edentulism. A recent cross-sectional study in Romania found that edentulism severity increased with age and was linked to poorer general health, particularly in individuals with cardiovascular and systemic conditions [40]. Similarly, a Polish survey reported that a history of CVD remained an independent predictor of tooth loss after adjusting for factors such as age and smoking [41]. Furthermore, a seven-year prospective cohort study observed a high prevalence of edentulism among cardiovascular patients undergoing full-arch implant rehabilitation, emphasizing the broader health implications in this population [42]. These findings support the relevance of complete edentulism as a potential marker for systemic health concerns, aligning with our observation of elevated edentulism prevalence among individuals with CVD in the U.S.

Although biological mediators were not measured directly in our dataset, several mechanistic pathways have been proposed to explain the bidirectional relationship between CVD and complete edentulism. One of the most plausible mechanisms underlying this association is chronic low-grade systemic inflammation, or “inflammaging,” a feature common to both periodontitis and atherosclerosis [43]. Elevated levels of inflammatory mediators such as C-reactive protein (CRP), interleukins (e.g., IL-6, IL-1β), tumor necrosis factor-alpha (TNF-α), and interferon-gamma (IFN-γ) have been reported in both conditions [44,45]. As the inflammatory cascade progresses, it may trigger the release of tissue-destructive enzymes and signaling molecules, including matrix metalloproteinases (MMPs), reactive oxygen species (ROS), and receptor activators of nuclear factor kappa-B ligand (RANKL) [44,45,46,47,48]. Together, these immune signals may contribute to vascular dysfunction and periodontal tissue breakdown [44,45,46,47,48]. While these pathways remain hypothetical in this context, they are supported by previous research linking systemic inflammation, periodontal disease, and cardiovascular outcomes [43,44,45,46,47,48].

Recent evidence indicates that systemic inflammation may persist even after complete tooth loss, partly due to chronic oral inflammatory stimuli such as denture-induced stomatitis, residual ridge resorption, and microbial colonization of prosthetic surfaces [49,50]. Removable dentures can act as reservoirs for *Candida albicans* and other pathogens, particularly in the presence of poor oral hygiene [49,50]. Mucosal atrophy resulting from ridge resorption may compromise the epithelial barrier function, which may increase mucosal permeability to toxins or pathogens [50,51]. Moreover, tooth loss has been associated with shifts in the oral microbiome, often characterized by reduced diversity and greater colonization by pro-inflammatory species [52,53]. These microbial changes may extend beyond the oral cavity and influence the gut microbiome via the “oral–gut axis,” potentially amplifying systemic inflammatory responses [52,53].

Immune dysregulation may further explain this association [54,55,56]. Patients with CVD may exhibit impaired neutrophil chemotaxis, reduced phagocytic activity, and abnormal oxidative responses [54,55,56]. These changes may diminish the host’s ability to clear pathogenic biofilms from the gingival sulcus and maintain a chronic inflammatory state in periodontal tissues [54,55,56]. Beyond inflammatory pathways, CVD is also associated with impaired vascular perfusion and endothelial damage, which may reduce blood flow to the gingiva and periodontium [57,58]. This limited circulation restricts oxygen and nutrient delivery to the periodontium, which may delay tissue repair and healing [57,58]. As a result, the periodontium may become more vulnerable to breakdown, setting the stage for tooth loss [57,58].

According to the current literature, periodontal pathogens such as *Porphyromonas gingivalis* can invade endothelial cells, induce dysfunction, and trigger pro-atherogenic responses, thereby promoting the development and progression of atherosclerotic lesions [59,60,61]. Studies reported that activities such as tooth brushing, debridement, or scaling could facilitate the entry of oral pathogens and their virulence factors into the bloodstream [59,60,61]. Moreover, *P. gingivalis* DNA and antigens can be detected in atherosclerotic plaque [59,60,61]. Recent findings also indicate that the presence of periodontal bacteria in the bloodstream or in situ within vascular lesions is associated with an increased risk of aneurysmal disease [59,60,61].

Although our analysis did not include data on xerostomia or medication use, previous studies suggest that antihypertensive agents, particularly calcium channel blockers, diuretics, and beta-blockers, may reduce salivary flow [62,63]. Xerostomia may result from anticholinergic effects or diuretic-induced dehydration [62,63]. Chronic dry mouth may impair the natural cleansing of the oral cavity, promote plaque accumulation, and elevate the risk of dental caries and periodontal disease [62,63].

**Figure 2:**
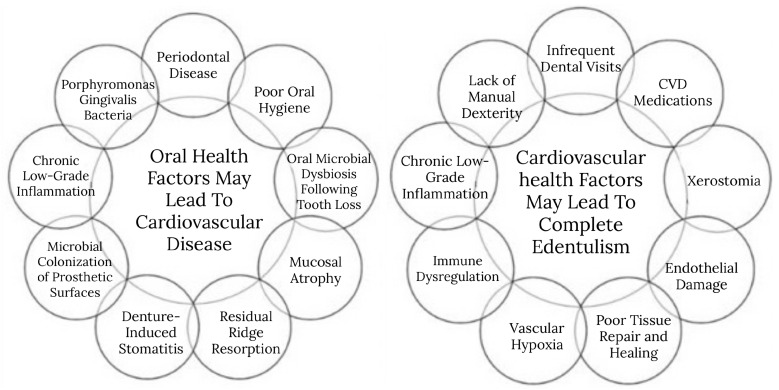
Proposed bidirectional pathways between cardiovascular and oral health. Note: The causal relationship cannot be inferred from this cross-sectional figure.

Behavioral and access-related factors may also mediate this relationship. Research has found that individuals with CVD may face barriers to maintaining regular dental care [64,65]. These may include physical limitations, financial burdens, competing health priorities, and medication burdens [64,65]. For example, some patients with heart conditions may avoid elective dental procedures due to concerns about bleeding risks from anticoagulant therapy or fear of triggering cardiac complications during dental treatment [66,67]. Additionally, behavioral risk factors—such as smoking, poor diet, lack of manual dexterity, particularly in older adults, may compound these challenges [68]. In such contexts, oral hygiene routines may decline, which may lead to plaque accumulation, periodontal disease progression, and eventual tooth loss [68,69]. The proposed bidirectional pathways between cardiovascular disease and oral health are illustrated in Figure 2.

Collectively, these findings support a multifactorial, synergistic relationship between CVD and complete edentulism. Shared inflammatory pathways, immune dysfunction, impaired vascular perfusion, medication side effects, and behavioral determinants may likely interact to increase the risk of complete edentulism among individuals with CVD. Recognizing these overlapping mechanisms emphasizes the need for integrated medical-dental care models, particularly for patients with chronic diseases.

### 4.1. Limitations of Research

Despite its strengths, this study has several limitations. First, the cross-sectional design precludes establishing a temporal sequence between CVD and complete edentulism; therefore, causal inferences cannot be drawn.

Second, important confounders—such as smoking status, oral hygiene practices, access to dental care, and medication use—were not included in the adjusted models. Smoking variables were available in both NHANES cycles but were excluded to maintain model parsimony. Measures of oral hygiene behaviors and dental service utilization were inconsistently collected across the 2015–2016 and 2017–2018 cycles or were limited to specific age groups. Medication use data were available but would have required extensive classification of drug types, including xerostomia-inducing agents, which was beyond the scope of this analysis. Consequently, residual confounding cannot be ruled out, and future studies should incorporate these variables to clarify the multifactorial pathways linking cardiovascular health and tooth loss.

Third, CVD status was self-reported, which may introduce a recall or misclassification bias. Although NHANES uses standardized questionnaires, self-reported data lack the clinical precision of objective diagnostic measures. Lastly, while dental examination data reliably identify edentulism, NHANES does not specify the underlying cause of tooth loss (e.g., periodontal disease vs. trauma), limiting interpretation of mechanistic pathways.

This study has notable strengths. Using data from the National Health and Nutrition Examination Survey (NHANES) enhances this study’s external validity, as NHANES employs a large, nationally representative sample of U.S. adults and includes both clinical dental assessments and self-reported cardiovascular outcomes. The inclusion of multiple CVD subtypes (heart attack, coronary heart disease, congestive heart failure, and stroke) provides a nuanced understanding of how different cardiovascular conditions are associated with complete tooth loss. Furthermore, the analysis adjusts for a broad range of demographic, socioeconomic, and health-related confounders.

### 4.2. Future Perspectives

Future research should build on these findings by utilizing longitudinal study designs to determine causal relationships between CVD and edentulism. Prospective cohort studies could help determine the temporal sequence and clarify whether tooth loss contributes to cardiovascular risk or vice versa. Incorporating biomarkers of inflammation, comprehensive oral health histories, and objective cardiovascular assessments would enable more precise exploration of underlying biological mechanisms. Future analyses should also include variables such as smoking status, oral hygiene behaviors, access to dental care, and medication use to capture the multifactorial nature of the oral–systemic health connection. From a clinical standpoint, interdisciplinary strategies that integrate dental evaluations into cardiovascular risk assessments could enhance early identification of at-risk individuals. Public health policies should also emphasize the importance of maintaining oral health as a preventive measure against systemic diseases, particularly among underserved and aging populations.

## 5. Conclusions

This study demonstrates a positive association between CVD and complete edentulism among U.S. adults. Individuals with CVD were more likely to be completely edentulous, and this association persisted across major subtypes, including myocardial infarction, coronary heart disease, congestive heart failure, and stroke. These findings add to the growing body of evidence linking oral and cardiovascular health through shared biological mechanisms such as systemic inflammation, immune dysregulation, and vascular dysfunction. Recognizing edentulism not only as a dental outcome but also as a potential marker of systemic disease burden may strengthen both clinical and public health strategies.

For clinicians, these results underscore the value of including oral health assessments, particularly edentulism, in comprehensive cardiovascular risk evaluations. Early referral to dental care providers could aid in prevention and promote holistic care. For policymakers, the findings highlight the urgent need to integrate oral health into national chronic disease prevention frameworks. Expanding access to dental services for high-risk groups, such as older adults and low-income populations, may help reduce the dual burden of tooth loss and CVD.

While this study focused on CVD as a predictor, the bidirectional interplay between oral and systemic health warrants ongoing investigation. Future longitudinal studies are needed to clarify causal pathways, determine temporal relationships, and evaluate whether interventions targeting oral health can lower cardiovascular risk. Such evidence could inform both clinical guidelines and health policy, enhancing interdisciplinary collaboration between medical and dental professionals.

## Figures and Tables

**Table 1:** Descriptive summary of population characteristics.

Independent Variable		With Dentition (*n*)	%	Complete Tooth Loss (*n*)	%	Total (*n*)	*p* Value
Heart Disease *	No heart disease	8583	84.5	1378	6.42	9961	<0.0001
	Have heart disease	941	7.33	385	1.76	1326	
Heart Attack (Myocardial Infarction (MI))	Yes	356	2.78	165	0.78	521	<0.0001
	No	9155	89.06	1593	7.42	10,748	
Coronary Heart Disease (CHD)	Yes	349	3.08	160	0.74	509	<0.0001
	No	9147	88.78	1590	7.39	10,737	
Congestive Heart Failure (CHF)	Yes	271	1.8	144	0.58	415	<0.0001
	No	9229	90.02	1616	7.61	10,845	
Stroke	Yes	346	2.37	136	0.64	482	<0.0001
	No	9167	89.46	1624	7.52	10,791	
Gender	Male	6963	42.21	2486	6.65	9449	0.5345
	Female	7329	44.36	2447	6.78	9776	
Race/Ethnicity	Hispanic	4065	15.14	1351	2.72	5416	0.00017
	White	4474	52.46	1742	7.41	6216	
	Black	3221	10.22	1023	1.67	4244	
	Asian	1747	4.83	463	0.75	2210	
	Others	785	3.92	354	0.87	1139	
Age (in years)	19–44	4345	32.54	455	1.38	4800	<0.0001
	45–59	2376	18.66	339	1.56	2715	
	Above 60	3061	18.07	990	3.14	4051	
Education Level	0–11	1931	10.8	550	2.13	2481	<0.0001
	HS/Ged	2118	21.46	443	2.48	2561	
	>HS	5466	59.58	762	3.55	6228	
Ratio of Family	<100%FPL	2889	69.58	1263	30.42	4152	
	100–99%FPL	3564	74.46	1222	25.54	4786	<0.0001
	200–399%FPL	3382	77.18	1000	22.82	4382	
	400% + FPL	2886	79.68	736	20.32	3622	
BMI	Underweight	1877	7.61	1288	4.39	3165	<0.0001
	Normal	4268	26.16	453	1.97	4721	
	Overweight	3648	24.58	409	2.09	4057	
	Obese	4355	30.68	463	2.52	4818	
Diabetes	Yes	1365	7.38	384	1.24	1749	0.0976
	No	12,647	78.54	3737	10.99	16,384	
	Borderline	273	1.67	58	0.17	331	

* Heart Disease includes one or more of the following: CHD, MI, CHF, and stroke. Note: Table 1 presents descriptive characteristics of the full 2015–2018 NHANES sample, including participants younger than 30 years. However, only adults aged ≥30 years are included in the Poisson regression models examining the association between CVD and complete edentulism.

**Table 2:** Multiple Poisson regression model for the association between heart attack and complete edentulism.

		Composite		
Covariate	Prevalence Ratio	Confidence Interval		*p* Value
		Lower	Upper	
Heart attack (MI) *	1.55	1.225	1.980	0.001
Sex (reference: male)	1.03	0.878	1.224	0.657
Age (reference: 30–44)				
45–59	2.10	1.558	2.848	<0.001
>60	3.70	2.576	5.326	<0.001
Education (reference: <high school)				
High school/GED	0.70	0.580	0.859	0.001
>High school	0.46	0.380	0.556	<0.001
Poverty (reference: <100% FPL)				
100–199% FPL	0.93	0.740	1.178	0.554
200–399% FPL	0.58	0.427	0.809	0.002
400% + FPL	0.42	0.294	0.609	<0.001
Race (reference: Hispanic)				
Non-Hispanic White	1.44	1.045	2.009	0.027
Non-Hispanic Black	1.50	1.109	2.051	0.010
Non-Hispanic Asian	1.77	1.198	2.624	0.006
Other	2.17	1.448	3.264	<0.001
Diabetes (reference: No Diabetes)				
Yes	1.10	0.884	1.385	0.361
Borderline	1.09	0.729	1.654	0.641
BMI (reference: Underweight)				
Normal	0.75	0.298	1.893	0.533
Overweight	0.86	0.333	2.251	0.762
Obese	0.78	0.316	1.955	0.594

* MI, myocardial infarction.

**Table 3:** Multiple Poisson regression model for the association between heart disease and complete edentulism.

		Composite		
Covariate	Prevalence Ratio	Confidence Interval		*p* Value
		Lower	Upper	
Heart Disease *	1.53	1.280	1.842	<0.001
Sex (reference: male)	1.03	0.879	1.215	0.676
Age (reference: 30–44)				
45–59	2.07	1.537	2.796	<0.001
>60	3.52	2.458	5.052	<0.001
Education (reference: <high school)				
High school/GED	0.71	0.582	0.866	0.001
>High school	0.46	0.382	0.559	<0.001
Poverty (reference: <100% FPL)				
100–199% FPL	0.93	0.741	1.168	0.554
200–399% FPL	0.59	0.431	0.813	0.002
400% + FPL	0.43	0.301	0.619	<0.001
Race (reference: Hispanic)				
Non-Hispanic White	1.41	1.016	1.962	0.040
Non-Hispanic Black	1.46	1.073	1.997	0.018
Non-Hispanic Asian	1.76	1.189	2.629	0.006
Other	2.14	1.413	3.242	0.001
Diabetes (reference: No Diabetes)				
Yes	1.06	0.851	1.335	0.562
Borderline	1.04	0.698	1.575	0.812
BMI (reference: Underweight)				
Normal	0.75	0.298	1.885	0.529
Overweight	0.85	0.329	2.232	0.746
Obese	0.77	0.313	1.915	0.569

* Heart Disease includes one or more of the following: CHD, MI, CHF, and stroke.

**Table 4:** Multiple Poisson regression model for the association between coronary heart disease and complete edentulism.

		Composite		
Covariate	Prevalence Ratio	Confidence Interval		*p* Value
		Lower	Upper	
Coronary Heart Disease	1.44	1.132	1.853	0.004
Sex (reference: male)	1.02	0.872	1.210	0.739
Age (reference: 30–44)				
45–59	2.12	1.572	2.872	<0.001
>60	3.73	2.598	5.75	<0.001
Education (reference: <high school)				
High school/GED	0.71	0.585	0.884	0.003
>High school	0.46	0.378	0.567	<0.001
Poverty (reference: <100% FPL)				
100–199% FPL	0.94	0.748	1.184	0.595
200–399% FPL	0.58	0.425	0.809	0.002
400% + FPL	0.42	0.294	0.605	<0.001
Race (reference: Hispanic)				
Non-Hispanic White	1.43	1.025	2.001	0.036
Non-Hispanic Black	1.51	1.110	2.066	0.010
Non-Hispanic Asian	1.77	1.199	2.618	0.005
Other	2.22	1.468	3.356	<0.001
Diabetes (reference: No Diabetes)				
Yes	1.11	0.877	1.408	0.367
Borderline	1.05	0.697	1.610	0.779
BMI (reference: Underweight)				
Normal	0.74	0.296	1.890	0.528
Overweight	0.85	0.325	2.225	0.734
Obese	0.78	0.315	1.959	0.596

**Table 5:** Multiple Poisson regression model for the association between congestive heart failure and complete edentulism.

		Composite		
Covariate	Prevalence Ratio	Confidence Interval		*p* Value
		Lower	Upper	
Congestive Heart Failure	1.58	1.216	2.067	0.001
Sex (reference: male)	1.01	0.862	1.193	0.858
Age (reference: 30–44)				
45–59	2.12	1.577	2.868	<0.001
>60	3.78	2.612	5.482	<0.001
Education (reference: <high school)				
High school/GED	0.70	0.574	0.864	0.001
>High school	0.45	0.374	0.559	<0.001
Poverty (reference: <100% FPL)				
100–199% FPL	0.93	0.739	1.169	0.523
200–399% FPL	0.58	0.425	0.809	0.002
400% + FPL	0.42	0.295	0.607	<0.001
Race (reference: Hispanic)				
Non-Hispanic White	1.45	1.041	2.019	0.029
Non-Hispanic Black	1.48	1.087	2.026	0.015
Non-Hispanic Asian	1.75	1.185	2.602	0.007
Other	2.25	1.494	3.407	<0.001
Diabetes (reference: No Diabetes)				
Yes	1.10	0.872	1.391	0.403
Borderline	1.04	0.690	1.578	0.831
BMI (reference: Underweight)				
Normal	0.75	0.299	1.910	0.544
Overweight	0.86	0.329	2.256	0.756
Obese	0.78	0.315	1.933	0.582

**Table 6:** Multiple Poisson regression model for the association between stroke and complete edentulism.

		Composite		
Covariate	Prevalence Ratio	Confidence Interval		*p* Value
		Lower	Upper	
Stroke	1.46	1.133	1.898	0.005
Sex (reference: male)	1.01	0.860	1.198	0.858
Age (reference: 30–44)				
45–59	2.13	1.583	2.872	<0.001
>60	3.80	2.636	5.486	<0.001
Education (reference: <high school)				
High school/GED	0.70	0.574	0.864	0.001
>High school	0.46	0.378	0.560	<0.001
Poverty (reference: <100% FPL)				
100–199% FPL	0.93	0.743	1.179	0.565
200–399% FPL	0.58	0.426	0.811	0.002
400% + FPL	0.42	0.296	0.607	<0.001
Race (reference: Hispanic)				
Non-Hispanic White	1.45	1.046	2.027	0.027
Non-Hispanic Black	1.49	1.095	2.047	0.013
Non-Hispanic Asian	1.78	1.204	2.646	0.005
Other	2.24	1.480	3.404	<0.001
Diabetes (reference: No Diabetes)				
Yes	1.10	0.878	1.384	0.387
Borderline	1.04	0.703	1.565	0.807
BMI (reference: Underweight)				
Normal	0.73	0.292	1.825	0.490
Overweight	0.83	0.322	2.162	0.701
Obese	0.76	0.310	1.898	0.556

**Table 7:** The prevalence rates among different CVD outcomes.

		Composite		
Covariate	Prevalence Ratio	Confidence Interval		*p* Value
		Lower	Upper	
Heart Disease *	1.53	1.280	1.842	<0.001
Heart Attack (MI)	1.55	0.878	1.224	0.657
Coronary Heart Disease	1.44	1.132	1.853	0.004
Congestive Heart Failure	1.58	1.216	2.067	0.001
Stroke	1.46	1.133	1.898	0.005

* Heart Disease includes one or more of the following: CHD, MI, CHF, and stroke.

## Data Availability

The data used in this article are publicly available and can be found at the Centers for Disease Control and Prevention (CDC) National Center for Health Statistics: National Health and Nutrition Examination Survey (NHANES) Questionnaires, Datasets, and Related Documentation, available at the following website: (https://wwwn.cdc.gov/nchs/nhanes/) accessed on 4 December 2024.

## Abbreviations

The following abbreviations are used in this manuscript:

CVD	Cardiovascular Disease
CHD	Coronary Heart Disease
MI	Myocardial Infarction
CHF	Congestive Heart Failure
